# Supplementary figures and images for: Identification and transcriptomic profiling of salinity stress response genes in colored wheat mutant
Source: PeerJ. 2024 Mar 6;12:e17043. doi: 10.7717/peerj.17043 (PMC10924784; doi:10.7717/peerj.17043)

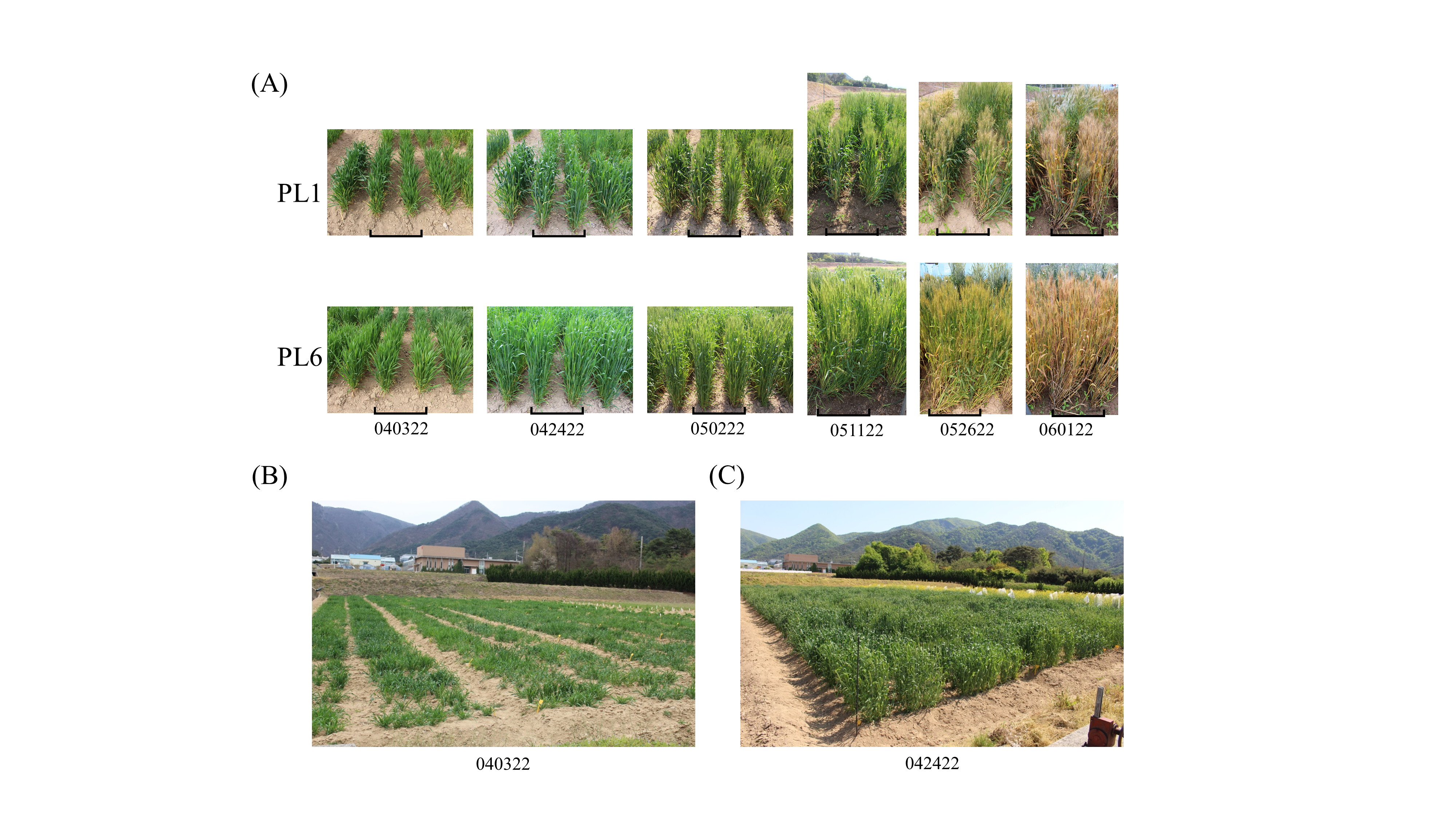

Supplement: Supplemental Information 1 [file peerj-12-17043-s001.tif]

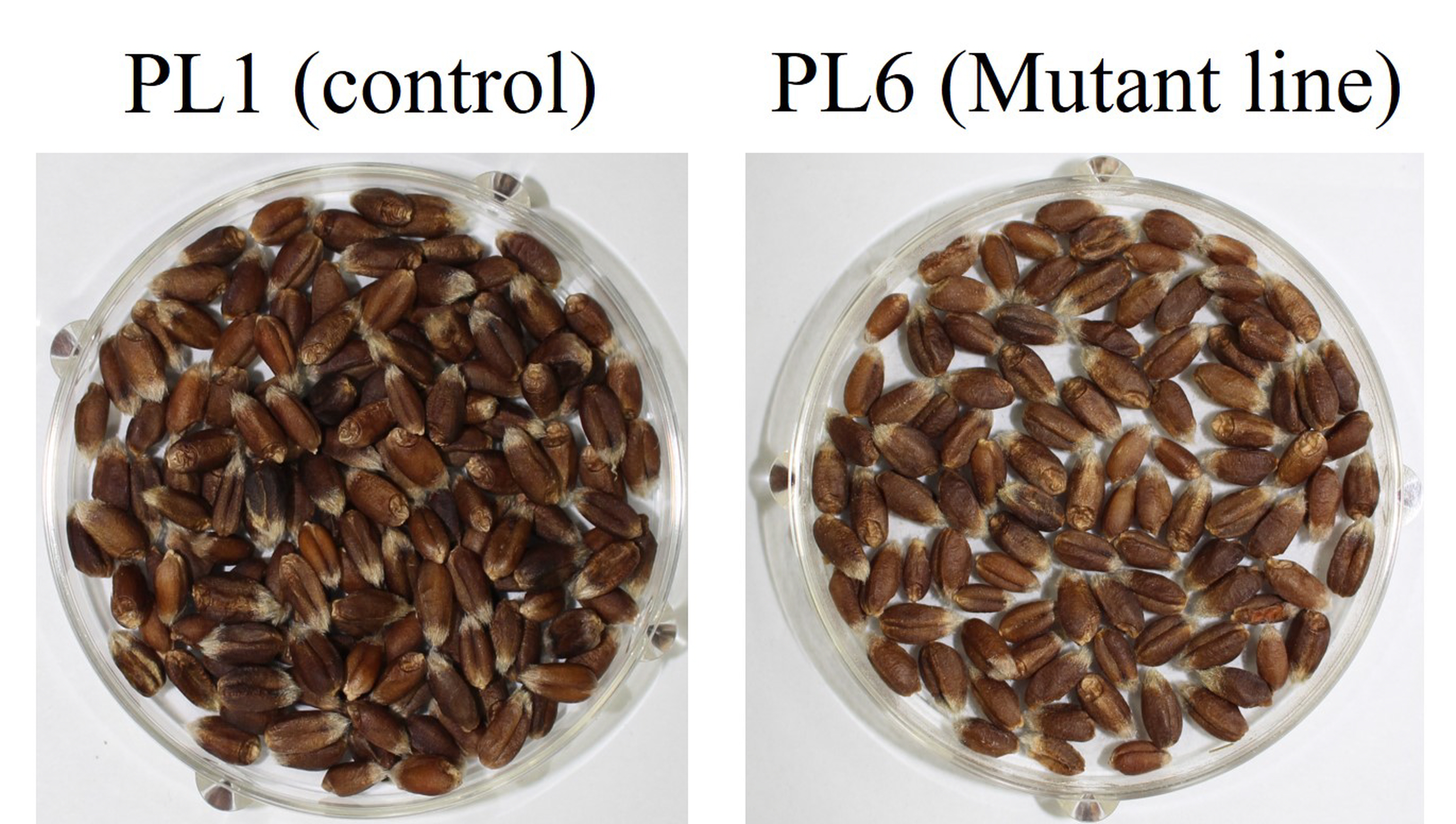

Supplement: Supplemental Information 2 — PL1 (control) and PL6 (mutant line) were used in this study. [file peerj-12-17043-s002.jpg]

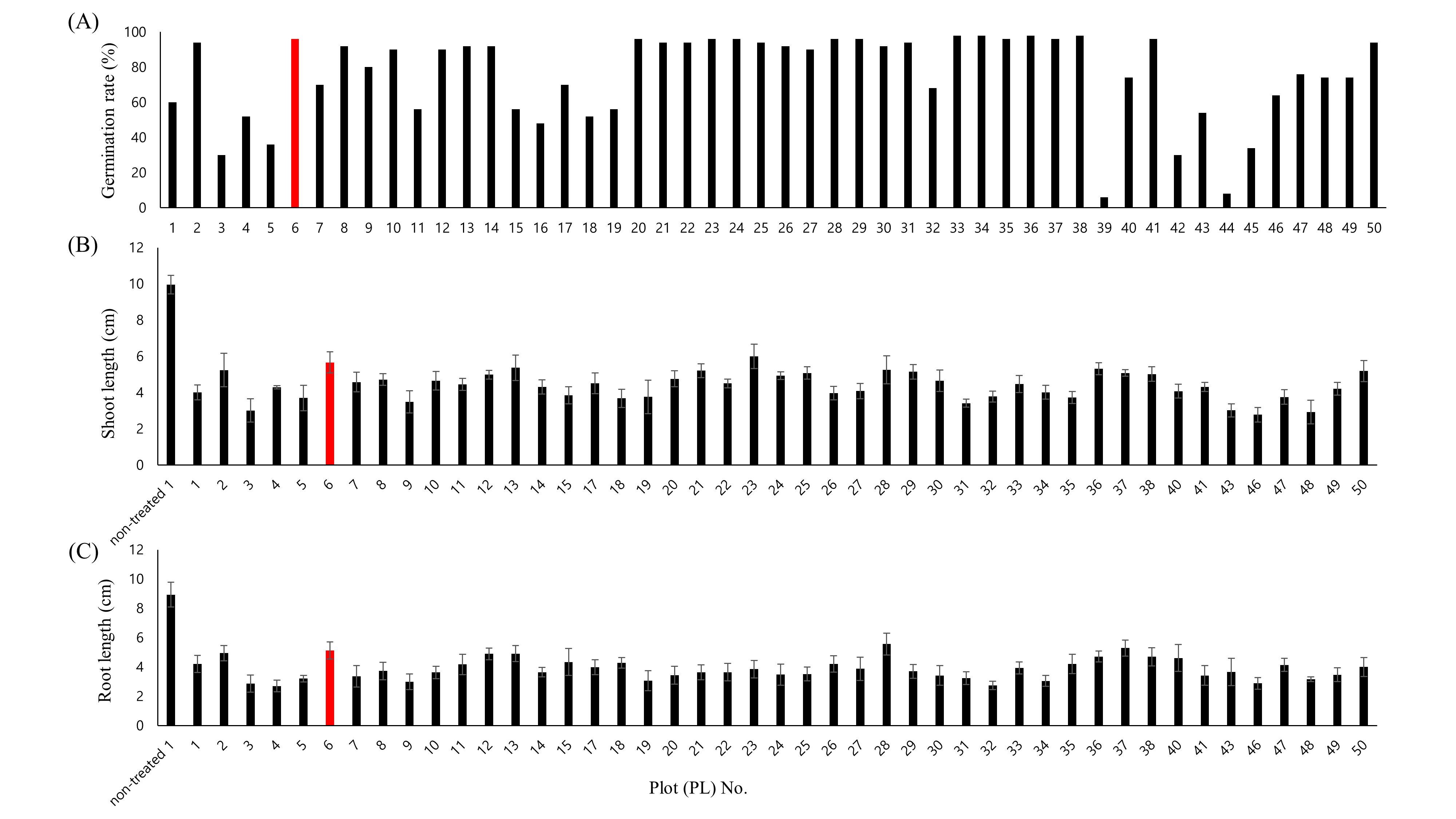

Supplement: Supplemental Information 3 — (A) Germination rate of mutant lines (PL2-PL49) and PL1 as the wild type control. (B) Shoot length of mutant lines (PL2-PL49) and PL1 as the wild type control. (C) Root length of mutant lines (PL2-PL49) and PL1 as the wild type control. For the preliminary screening of the selected mutant lines, 100 seeds from each line were placed in a phytohealth chamber (SPL Life Sciences) with two layers of germination paper, and a total volume of 200 ml of the solution containing 150 mM NaCl was applied to them at a temperature of 22° C. After 4 days, the germination rate, shoot length, and root length were recorded. PL1 served as the wild type control in these experiments. [file peerj-12-17043-s003.tif]
