# Supplementary material for: Identification and transcriptomic profiling of salinity stress response genes in colored wheat mutant
Source: PeerJ. 2024 Mar 6;12:e17043. doi: 10.7717/peerj.17043 (PMC10924784; doi:10.7717/peerj.17043)
Supplement: Supplemental Information 12 [file peerj-12-17043-s012.docx]

**Essential MIQE Checklist**

| **Experimental Design** | |  |
| --- | --- | --- |
| Definition of experimental and control groups | page5:156-157 | ∨ |
| Number within each group | page5:156-157, page11:350-360 | ∨ |
| **Sample** | |  |
| Description | page4:120-131 | ∨ |
| Microdissection or macrodissection | Not Applicable | ∨ |
| Processing procedure | page4:130-131 | ∨ |
| If frozen - how and how quickly? | page4:130-131 | ∨ |
| If fixed - with what, how quickly? | Not Applicable | ∨ |
| Sample storage conditions and duration (especially for FFPE samples) | page4:130-131 | ∨ |
| **Nucleic Acid Extraction** | |  |
| Procedure and/or instrumentation | page5-6:155-193 | ∨ |
| Name of kit and details of any modifications | page5-6:155-193 | ∨ |
| Details of DNase or RNAse treatment | page5:159-160 | ∨ |
| Contamination assessment (DNA or RNA) | page5:159-163 | ∨ |
| Nucleic acid quantification | page5:159-163 | ∨ |
| Instrument and method | page5:159-163 | ∨ |
| RNA integrity method/instrument | page5:159-163, and Table 1 | ∨ |
| RIN/RQI or Cq of 3' and 5' transcripts | page5:159-163, and Table 1 | ∨ |
| Table 1. RNA quality information   \| Sample Name \| Conc.(ng/ μL) \| Final Volume (μL) \| Total Amount (μg) \| RIN \| rRNA Ration \| \| --- \| --- \| --- \| --- \| --- \| --- \| \| PL1_0h \| 1618.106 \| 16 \| 25.89 \| 9.4 \| 1.6 \| \| PL1_3h \| 1511.103 \| 22 \| 33.244 \| 9 \| 1.6 \| \| PL1_24h \| 1577.114 \| 20 \| 31.4542 \| 9.2 \| 1.5 \| \| PL1_48h \| 1352.761 \| 18 \| 24.35 \| 9.2 \| 1.7 \| \| PL6_0h \| 2629.068 \| 15 \| 39.436 \| 9.3 \| 1.7 \| \| PL6_3h \| 1138.332 \| 20 \| 22.767 \| 8.7 \| 1.8 \| \| PL6_24h \| 1452.326 \| 16 \| 23.237 \| 9.2 \| 1.8 \| \| PL6_48h \| 2367.427 \| 19 \| 44.981 \| 9.1 \| 1.8 \| | |  |
| Inhibition testing (Cq dilutions, spike or other) | Not Applicable | ∨ |
| **Reverse Transcription** | |  |
| Complete reaction conditions | page6:186-193 | ∨ |
| Amount of RNA and reaction volume | page6:186-187 | ∨ |
| Priming oligonucleotide (if using GSP) and concentration | Oligo dT, page6:186-187 | ∨ |
| Reverse transcriptase and concentration | page6:186-187 | ∨ |
| Temperature and time: page6:186-187 | page6:186-187 | ∨ |
| **qPCR Target** | |  |
| Gene symbol | page10:350-360 | ∨ |
| Sequence accession number | *TRAESCS1B02G095800, TRAESCS1B02G038700, TRAESCS1B02G102200, TRAESCS1B02G138100, TRAESCS2A02G046200, TRAESCS1B02G048900, TRAESCS1B02G071800, TRAESCS7D02G246600, TRAESCS6B02G466700, TRAESCS6B02G017900, TRAESCS5D02G537600, TRAESCS1B02G105100* |  |
| Amplicon length | Table 2 | ∨ |
| *In silico* specificity screen (BLAST, etc) | Table 2 | ∨ |
| Table 2. Amplicon length of RT-qPCR products   \| Group \| Accession Number \| Blast description \| Amplicon length (bp) \| \| --- \| --- \| --- \| --- \| \| Cluster 1 \| *TRAESCS1B02G095800* \| peroxidase 2 \| 144 \| \| *TRAESCS1B02G038700* \| protein NRT1/ PTR FAMILY 6.2 \| 70 \| \| *TRAESCS1B02G138100* \| auxin-responsive protein IAA15 \| 159 \| \| *TRAESCS1B02G102200* \| replication protein A 70 kDa DNA-binding subunit C-like \| 143 \| \| Cluster 2 \| *TRAESCS2A02G046200* \| nuclear transport factor 2 (NTF2)-like protein \| 82 \| \| *TRAESCS1B02G048900* \| histone H2A \| 162 \| \| *TRAESCS1B02G071800* \| thylakoid membrane protein TERC, chloroplastic \| 99 \| \| *TRAESCS7D02G246600* \| probable histone H2A variant 3 \| 181 \| \| Cluster 3 \| *TRAESCS6B02G466700* \| protein argonaute 1C-like isoform X2 \| 121 \| \| *TRAESCS1B02G105100* \| ribosome biogenesis protein NOP53 \| 168 \| \| *TRAESCS6B02G017900* \| predicted protein \| 165 \| \| *TRAESCS5D02G537600* \| aspartokinase 1, chloroplastic-like \| 105 \| | |  |
| Location of each primer by exon or intron (if applicable) | exon | ∨ |
| What splice variants are targeted? | Not Applicable | ∨ |
| **qPCR Oligos** | |  |
| Primer sequences | Refer to Supplementary Table 1 (Table S1) | ∨ |
| Location and identity of any modifications | Not Applicable | ∨ |
| **qPCR Protocol** | |  |
| Reaction volume and amount of cDNA/DNA | Page6:188-193 | ∨ |
| Primer, (probe), Mg++ and dNTP concentrations | Page6:188-193 | ∨ |
| Polymerase identity and concentration | Page6:188-193 | ∨ |
| Buffer/kit identity and manufacturer | Page6:188-193 | ∨ |
| Additives (SYBR Green I, DMSO, etc.) | Page6:188-193 | ∨ |
| Complete thermocycling parameters | Page6:188-193 | ∨ |
| Manufacturer of qPCR instrument | Page6:190-192 (Bio-rad) | ∨ |
| **qPCR Validation** | |  |
| Specificity (gel, sequence, melt, or digest) | Melt | ∨ |
| For SYBR Green I, Cq of the NTC | NTC with a Cq less than 38 | ∨ |
| Standard curves with slope and y-intercept | Slope: -3.450, y-int:35.332 | ∨ |
| PCR efficiency calculated from slope | Efficiency less than 90  Efficiency greater than 110 | ∨ |
| r2 of standard curve | Std curve R^2^ less than 0.980 | ∨ |
| Linear dynamic range | Not Applicable | ∨ |
| Cq variation at lower limit | Not Applicable | ∨ |
| Evidence for limit of detection | Not Applicable | ∨ |
| If multiplex, efficiency and LOD of each assay. | Not Applicable | ∨ |
| **Data Analysis** | |  |
| qPCR analysis program (source, version) | Bio-rad CFX manager | ∨ |
| Cq method determination | The measured Cq values are proportional to the log base 2 (log2) of the concentration of the measured, which is a logarithmic response | ∨ |
| Outlier identification and disposition | Cq more than 3 sd from technical means (statistical outlier) | ∨ |
| Results of NTCs | Not Applicable | ∨ |
| Justification of number and choice of reference genes | Actin, encodes a major structural protein in many cell types and considered the ideal reference gene for RT-qPCR analysis and is most frequently used. Actin (AB181991) gene was used to normalize the quantification of expression  Page6:192-193 | ∨ |
| Description of normalisation method | Comparative Cq (△△Cq) method | ∨ |
| Number and stage (RT or qPCR) of technical replicates | *n* = 3, page25:927-932 | ∨ |
| Repeatability (intra-assay variation) | See error bars in Figure 8 | ∨ |
| Statistical methods for result significance | Independent Samples T-test | ∨ |
| Software (source, version) | IBM SPSS Statistics25 | ∨ |
